# Eyes on Memory: Pupillometry in Encoding and Retrieval

**DOI:** 10.3390/vision8020037

**Published:** 2024-06-14

**Authors:** Alex Kafkas

**Affiliations:** School of Health Sciences, Division of Psychology, Communication and Human Neuroscience, University of Manchester, Manchester M13 9PL, UK; alexandros.kafkas@manchester.ac.uk

**Keywords:** pupil response, episodic memory, neurotransmission, encoding, novelty, familiarity, recollection, false memory

## Abstract

This review critically examines the contributions of pupillometry to memory research, primarily focusing on its enhancement of our understanding of memory encoding and retrieval mechanisms mainly investigated with the recognition memory paradigm. The evidence supports a close link between pupil response and memory formation, notably influenced by the type of novelty detected. This proposal reconciles inconsistencies in the literature regarding pupil response patterns that may predict successful memory formation, and highlights important implications for encoding mechanisms. The review also discusses the pupil old/new effect and its significance in the context of recollection and in reflecting brain signals related to familiarity or novelty detection. Additionally, the capacity of pupil response to serve as a true memory signal and to distinguish between true and false memories is evaluated. The evidence provides insights into the nature of false memories and offers a novel understanding of the cognitive mechanisms involved in memory distortions. When integrated with rigorous experimental design, pupillometry can significantly refine theoretical models of memory encoding and retrieval. Furthermore, combining pupillometry with neuroimaging and pharmacological interventions is identified as a promising direction for future research.

## 1. Introduction

Over the past decade, pupillometry has risen as an indicator of long-term memory, with an increasing number of research studies using this methodology. In this review, I will explore how pupillometry has contributed to our understanding of memory formation and retrieval processes especially in the context of recognition memory. I will discuss recent findings that demonstrate pupillometry’s role in memory research, detailing the specific conditions under which it can distinctly assess memory functions. Furthermore, the discussion will cover methodological factors influencing pupil response and the strategies for isolating ‘pupil memory signals’ by experimentally managing these variables. This review does not aim to be exhaustive and mainly focuses on studies using recognition memory, although it includes relevant studies using recall. The recognition memory paradigm provides more control over the retrieval process, allowing for important conclusions regarding old/new effects and the processes supporting memory outcomes.

## 2. Autonomic and Central Nervous System Control of Pupil Response

Before considering what pupillometry has contributed so far to our understanding of memory encoding and retrieval mechanisms, it is relevant to consider the anatomy of pupil response as this can have implications for the interpretation of findings. Pupil size is regulated by the autonomic nervous system. The process of pupil dilation and constriction is managed by two opposing muscle groups in the iris: the circular or sphincter muscles, controlled by the parasympathetic nervous system via cholinergic neurotransmission, leading to constriction. On the other hand, the radial muscles, or dilator muscles are responsible for dilation under the influence of the sympathetic nervous system [1]. Pupil dilation can result directly from sympathetic stimulation of the dilator muscle or through the suppression of the sphincter muscle by the parasympathetic system [2]. The sympathetic control of pupil response originates in the hypothalamus, whereas the parasympathetic innervation originates at the Edinger–Westphal nucleus of the midbrain. Nevertheless, both autonomic pathways mediating pupillary responses hold reciprocal connections with central nervous system (CNS) structures and nuclei [3]. This bidirectional communication allows the autonomic nervous system to modulate, and be modulated by, cognitive processes. This highlights a complex interplay between physiological responses and cognitive states.

Increasing evidence has linked fluctuations in pupil size with differential neurotransmission in the brain [4,5]. This indicates that pupillometry can be a sensitive tool for tracking the activity of various neurotransmitter systems. The parasympathetic nervous system, as primarily cholinergic, uses acetylcholine to communicate with cortical and subcortical brain structures and has been linked to learning and neural plasticity changes due to memory encoding [6,7,8]. Also, reduced cholinergic input to the limbic system via the basal forebrain has been linked to deficits in long-term memory encoding [9,10], and memory dysfunction in Alzheimer’s disease [11]. The sympathetic system is noradrenergic, while pupil dilation changes have been linked to activity in locus coeruleus (LC) [12,13], which is a hotspot of noradrenergic [14,15] and dopaminergic [16,17] input to the limbic system and the hippocampus [18]. These observations provide a clear link between pupil response patterns, and differential neurotransmission which may accompany, as we will examine below, memory processes and retrieval outcomes.

## 3. Novelty Detection and Encoding Mechanisms

Novelty is inherently motivating, since encountering new information could signal an important change in our surroundings that merits remembering and integrating with what we already know [19]. This could be for the purpose of avoiding potential dangers or capitalizing on possible benefits in the future. Nonetheless, the notion of novelty is multifaceted, implying that various forms of novelty exist, each potentially affecting how we process, internalize, respond to, and recall or recognize these novel encounters later on [20,21]. Hence, the type of novelty can play a significant role in the learning process and the kind of memory that underpins the subsequent retrieval of new information. The positive impact of novelty on memory enhancement is not guaranteed, as it does not consistently result in improved memory retention (see e.g., [22,23,24]). This suggests that memory may prioritize some types of novel information under specific circumstances, but not universally. Consequently, identifying the specific circumstances that lead to this enhancement necessitates systematic investigation using various methodological tools.

The pupil response patterns during novelty detection and ensuing memory encoding can have implications and may allow exploring the contribution of different encoding mechanisms. At the same time, recent proposals [5,25] have underscored the value of analyzing pupil response patterns to gain insights into the brain’s pathways that play a crucial role in cognitive processes, such as memory encoding [21]. It has been demonstrated that pupil responses at the time of encoding might predict the type and strength of future memory [26,27,28,29,30,31]. However, there is lack of consistency across studies regarding the specific pupil response patterns that predict later memory performance. Previous research has shown that increased pupil dilation is associated with stimuli that are remembered as opposed to those that are forgotten (e.g., [28,31,32,33]), especially in cases of contextual or unexpected novelty [32]. One interpretation of these findings is that resource allocation associated with processing of events is reflected in pupil dilation patterns [34,35] and subsequently predicts the strength of memory. Nevertheless, other findings indicate that decreased pupil dilation, or pupil constriction, may predict the type and/or intensity of subsequent memory [26,29,30,36]. This is in line with the pupil old/new effect, where diminished pupil dilation is observed for new compared to old stimuli in recognition memory tasks ([37,38,39]; see also Section 3 below).

This discrepancy in the shape of pupil patterns predicting subsequent memory has also been found when negative stimuli are encoded. A recent study [40] highlighted that the incidental encoding of both emotional and neutral stimuli led to pupil constriction patterns predictive of subsequent memory. This is similar to the previous findings with incidental and expected novelty [26,41]. The effect was particularly more pronounced in the case of negatively valanced stimuli, which may be attributed to parasympathetically controlled attentional focus to potentially threatening stimuli. These pupillometric findings imply that physiological responses to emotionally charged or threatening stimuli, manifested through pupil constriction, not only prepare the organism for potential threats, but also enhance our ability to remember these stimuli. However, another study [33], involving negative and neutral stimuli at encoding, found that increased pupil dilation predicted recall accuracy for negative stimuli. This study used an intentional encoding task coupled with a free recall retrieval paradigm. Therefore, the encoding task seems to play a crucial role in how emotional stimuli are processed and may influence the encoding process, as will be discussed below.

### 3.1. Pupil Response Patterns at Encoding Depend on the Type of Novelty Detected

Collectively, the inconsistent findings related to the shape of pupil response patterns signaling successful memory formation, indicate that novelty detection, and ensuing memory encoding, draw on different mechanisms depending on experimental factors that modulate the type of novelty detected. In other words, we may assume that novelty effects in memory formation extend beyond a uniform response to all new information, but take into account the type of novelty detected. Indeed, a variety of theoretical frameworks have been proposed to distinguish between different types of novelty. These have primarily focused on the distinctions between contextual and stimulus novelty [20,42]. Contextual novelty refers to the introduction of new combinations of stimuli within a novel context, whereas stimulus novelty highlights the introduction of a stimulus that has not been previously encountered. More recently, theories have begun to consider the role of expectation—or the predictability of an experience—as a crucial determinant of novelty [21]. According to this perspective, novelty can be categorized into four principal types: contextual, associative, stimulus, and pure/semantic novelty. Importantly, contextual novelty pertains to the unexpected combination of familiar and new elements within a specific context. In contrast, stimulus novelty concerns the introduction of new elements that are more or less expected and certainly do not stand out as odd or unexpected occurrences.

Previous behavioral studies have shown that novel unexpected or surprising stimuli lead to increased subsequent recollection, while those that are expected result in an enhanced sense of familiarity. Familiarity refers to recognizing an item by itself, lacking the retrieval of specific contextual details linked to its previous encounter. On the other hand, recollection involves recalling detailed associative information linked to the stimulus previous encounter at an encoding episode [38,43,44,45]. This previous evidence highlights that the encoding process is affected by the type of novelty and may lead to the formation of different types of memory. These memory types may vary in the degree of associative detail they involve. This proposal was directly investigated in a recent study [41] grounded in the concept that novelty, whether expected or unexpected, plays a crucial role in the memory processes involved at encoding.

This study systematically manipulated the expectancy of novelty and its impact on memory encoding and pupil response patterns. Participants underwent a rule learning task to establish contingency relationships between symbols and the type of subsequent stimulus (either man-made or natural). This task aimed to create strong expectations regarding the symbol–stimulus sequences. In the encoding phase, stimuli were presented as either expected/conforming to the learned association or unexpected/violating the learned association. Participants’ pupil responses were recorded at this phase and their memory for the encoded stimuli was tested afterwards. The retrieval task used a recognition memory paradigm, in which participants could classify the presented stimuli as new, familiar or recollected.

#### The Effect of Expectation

**Figure 1:**
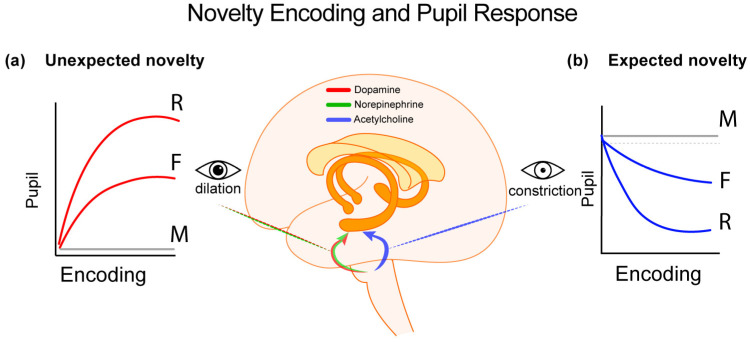
Differential pupillary responses to novelty, their brain basis and the different behavioral outputs. (**a**) Contextual novelty, characterized by the unexpected appearance of new events, triggers dopaminergic and noradrenergic signals to the hippocampus and parahippocampal gyrus. Significant dopaminergic contributions to the medial temporal lobe come from the midbrain—specifically the locus coeruleus and the substantia nigra/ventral tegmental area [32,47]. Their roles are evident in the sympathetic control of pupil dilation, where greater dilation correlates with enhanced memory formation [41]. (**b**) In contrast, absolute stimulus or expected novelty, engages cholinergic inputs to the medial temporal lobe originating from the pedunculopontine nucleus and basal forebrain. Their impact appears to drive the parasympathetically mediated pupil constriction patterns, while the extent of pupil constriction is predictive of stronger memory formation [41]. M = missed/forgotten stimuli; F = familiar; R = recollected stimuli.

This study found distinct effects of expectancy on memory encoding, importantly, showing that different pupil response patterns at encoding are modulated by the expectedness of the encoded stimuli (Figure 1). Specifically, increased pupil dilation was observed for unexpected stimuli. This pupil pattern was predictive of the strength and type of subsequent memory, with increased dilation predicting stronger subsequent familiarity and recollection. This suggests heightened attentional and cognitive processing for stimuli that violate prior expectations. On the other hand, decreased pupil response (constriction) was associated with expected stimuli. Critically, the degree of constriction was predictive of memory strength and type. In other words, increased constriction patterns predicted stronger familiarity and recollection. These findings highlight, therefore, the critical role of expectancy in modulating memory encoding, as evidenced by pupil response patterns. The differential pupil responses—dilation for unexpected novelty and constriction for expected novelty—suggests the engagement of distinct neural pathways at encoding influenced by the stimulus’s expectancy. In other words, pupil response patterns serve as indicators of the underlying cognitive processes during memory formation. This suggests that the brain employs different encoding strategies based on the novelty type.

To account for the inconsistencies in the previous literature related to the shape of pupil patterns that predict subsequent memory, it can be proposed [20,21] that different neural pathways and neurotransmitter systems are tuned in to different types of novelty. Specifically, contextual, surprising and unexpected novelty modulates the noradrenergic sympathetic system resulting in dopamine and norepinephrine release in the hippocampus–the brain region that supports deeper (e.g., associative) memory formation [46]. This is accomplished via input from LC [18] and results pupil dilation. On the other hand, according to the same account, detection of absolute and expected novelty modulates the cholinergic parasympathetic system. This results in acetylcholine release in the hippocampus, via the basal forebrain, leading to pupil constriction patterns (see Figure 1).

### 3.2. The Encoding Task as a Confound

A methodological factor that should be considered is the type of task participants are asked to complete and the intentionality of the encoding task. It can be argued that an intentional encoding paradigm, coupled with a decisional task, would actually infiltrate the pupil response related to encoding. For example, in a recent study [48], reward anticipation on the success of subsequent memory was explored. While reward anticipation was linked to increased pupil dilation, smaller dilation during this anticipation phase correlated with improved memory success. However, no definitive connection was established between pupil response at encoding and later memory performance. The authors suggested that the complexity of their task, which involved making precise categorical decisions and executing manual responses, might have obscured any potential effects. They highlighted that the mental and motor demands of the task, known to influence pupil size, along with the added complexity introduced by monetary incentives (to manipulate reward), could have concealed any direct links between pupil size atencoding and subsequent memory outcomes.

Indeed, in the previously discussed study [41], a free-viewing task was chosen for the encoding phase, while expectation was manipulated based on prior learned contingencies. Given the well-documented sensitivity of pupil response to intensive cognitive engagement and the demands on working memory [49], it is clear that employing a decision-making task could influence pupil dynamics. These potential changes in pupil size may not directly signify the formation of memory for the stimulus but rather the cognitive and decisional processes involved. For example, it has been reported [50] that pupil response patterns at encoding do not serve as reliable predictors of subsequent memory. Instead, according to this prior study, they reflect the urgency associated with the decision-making process. However, attributing pupil response exclusively to the urgency of decision-making does not fully capture the effects of memory encoding, especially when such responses are observed in tasks designed to heighten decisional pressure. This perspective overlooks the fact that the cognitive effort and temporal constraints inherent to an encoding task could influence the pupil responses, potentially concealing the true impact of memory formation on pupil response.

In essence, the interplay between cognitive demand, time pressure during task performance, and their collective effect on pupil response complicates the direct attribution of pupil changes to memory encoding processes. This complexity suggests that experimental designs in pupillometry must account for, and as much as possible control, the interactions with other cognitive processes. Overall, the interplay between cognitive demand, decisional processes, and the stimulus’s novelty type must be considered to fully understand the mechanisms underlying memory formation.

## 4. Pupil Response at Retrieval

### 4.1. The Pupil Old/New Effect and Implications for the Episodic Memory System Development

Pupillometric findings can be used to shed light into the processes involved at retrieval, when participants engage in retrieving or recognizing previously encountered stimuli and discriminating them from novel ones. This approach, commonly known as the recognition memory paradigm, holds significant importance. It is relevant not only because we frequently need to determine whether we have previously encountered stimuli in our daily lives [44], but also because it enables the investigation of how various types of memory contribute to making recognition decisions. Correctly identified previously encountered items lead to higher pupil dilation than correctly identified new items. This phenomenon is known as the pupil old/new effect [32,37,38]. This finding has also been replicated when comparing familiar versus unfamiliar branded products [51] and when a continuous recognition memory task is used [52].

In another study [53], the authors explored whether infants and adults exhibit similar episodic memory when discriminating new and old stimuli. Adults showed larger pupil dilation to items they had previously encountered, and 7-month-old infants displayed a clear pupil old/new effect, suggesting the development of episodic memory. However, unlike adults who showed this effect after one presentation, infants required two presentations. This suggests an important developmental difference in memory consolidation. Unlike 7-month-olds and adults, 4-month-old infants did not show a significant old/new effect, indicating that the episodic memory functions observed in older infants and adults are not yet developed at this age. This study supports the idea of a developmental continuity in episodic memory starting from infancy. The use of pupillometry allowed the identification of a non-verbal indicator of recognition memory, which can be used with young children with limited verbal abilities.

### 4.2. Pupil Response and the Type of Memory

**Figure 2:**
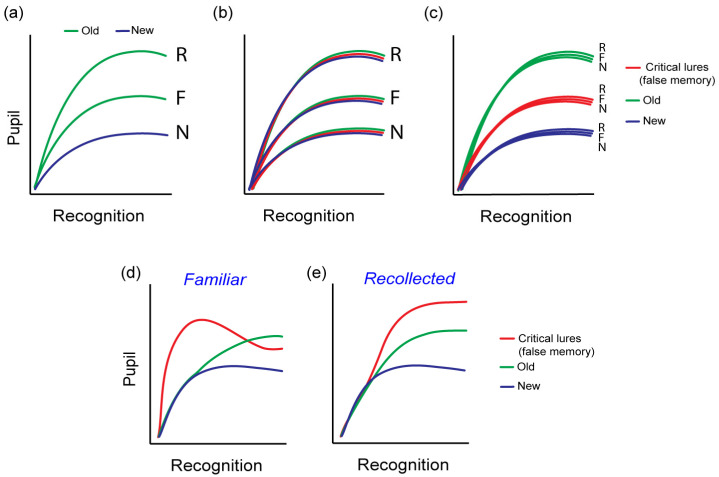
Pupil response patterns at retrieval. (**a**) The pupil old/new effect with accurately recognized old stimuli accompanied by increased pupil dilation. The memory type (F = familiar; R = recollected) further differentiates the pupil dilation pattern in accurate recognition [38]. (**b**,**c**) Hypothetical cause of the pupil old/new effect, (**b**) driven by subjective memory experience irrespective of true old/new status or (**c**) by objective old/new status irrespective of subjective memory response. (**d**,**e**) Pupil response discriminates true from false memories at different temporal stages during recognition memory decisions depending on the type of reported memory (data from [57]).

Pupil dilation varies among the types of memory supporting recognition memory decisions [38,39]. According to a widely accepted model of recognition memory, familiarity is identified as the recognition of an item as one previously encountered, whereas recollection is the process of recalling specific associative details from the moment of encoding [43,44,45,54]. Aligning with the pupil old/new effect, it has been observed that responses to new items cause the least pupil dilation, those based on familiarity cause a moderate increase in pupil dilation, while responses involving recollection cause the greatest degree of dilation (Figure 2a). This is the case even when strong familiarity and recollection are characterized by closely matched reported confidence and accuracy [38]. A more recent study [55] found that cues that allow greater successful recall of associated stimuli result in increased pupil dilation. More importantly, the extent of pupil dilation correlated with the amount of contextual detail recalled. Importantly, a prior study [56] also showed increased pupil dilation for spontaneously recalled words when using a free recall task. Thus, larger pupil dilation is associated with the retrieval of more detailed contextual information. Collectively, the pupil old/new findings and those differentiating familiarity-based from recollection-based recognition, indicate that the variation in pupil size signifies the successful retrieval of associative details from a specific episode and it is not just a reflection of memory strength. Thus, recollected or recalled stimuli are characterized by the retrieval of greater associative detail, greater engagement of the hippocampus and increased pupil dilation relative to strength-matched familiarity [38]. This finding appears to be independent of retrieval effort as the pupillometric differences between familiar and recollected stimuli have also been found even when recollection is spontaneous and effortless [27,38].

### 4.3. The Familiarity/Novelty Conundrum

Recollection or cued recall in the case of recall tasks cannot be the only driver for the pupil old/new effect. As noted above, marked differences have been found even when comparing correct identification of new stimuli (correct rejections) relative to those judged as familiar, even when recollected stimuli are excluded. This comparison was explored further in a study aimed to systematically explore the underlying causes of the pupillary old/new effect [27]. This was explored by controlling for traditional interpretations related to effort or decision complexity. Specifically, factors including targetness of old stimuli, effort, recollection-based retrieval, and complexity of recognition decisions were systematically controlled in a series of experiments. Participants were asked to discriminate new from old stimuli in two separate conditions emphasizing either novelty or familiarity detection. Recognition responses were provided using a rating scale that gauged perceived familiarity or novelty and instances of recollection. A further experiment modified the task to a simple ‘yes/no’ decision to explore the consistency of the pupillary effects for recognition tasks characterized by different degrees of complexity. Remarkably, both experiments yielded consistent results, namely pupil dilation was significantly larger for stimuli identified as familiar (excluding instances of recollection) compared to those deemed new, irrespective of the perceived targetness of familiarity or novelty.

Therefore, while the retrieval of contextual details (recollection or recall) can lead to increased pupil dilation, as mentioned previously, recognition based solely on pure familiarity also induces greater pupil dilation compared to new items identification. Additionally, it is crucial to highlight that these effects cannot be attributed to the effort or complexity involved in identifying familiar stimuli, as the two conditions emphasizing familiarity or novelty detection, led to matched accuracy and response times. Hence, the increased pupil dilation associated with familiar stimuli does not stem from variation in difficulty linked to distinguishing between familiarity and novelty. These findings suggest that the pupil dilation observed in recognition tasks is not a mere reflection of memory strength or the effort involved at retrieval. Instead, it is intricately linked to the cognitive processing related to distinguishing familiarity and novelty.

If the hypothesis of cognitive effort falls short of adequately explaining the pupil old/new effect, and the retrieval of contextual information alone does not account for this phenomenon, then what drives this effect? A plausible explanation might lie in the neural networks underlying the processes of familiarity and novelty detection. Drawing on a similar paradigm as in the study discussed above, an fMRI study by Kafkas and Montaldi [58] demonstrated that the brain signals familiarity and novelty through two distinct, yet partially overlapping, neural networks responsible for identifying familiar and new stimuli, respectively. Therefore, the differential pupil responses observed for old and new stimuli could be indicative of the different cognitive processes for the detection of familiarity and novelty, supported by unique yet intersecting neural pathways. Therefore, the organization of the memory system, which distinctly handles signals of familiarity and novelty, facilitates the brain’s capacity to recognize and assess novelty and familiarity. This organization not only aids in the retrieval of previously stored information, but also enhances the encoding of new stimuli.

## 5. Is the Pupil Old/New Effect a True Memory Signal: The Case of False Memories

A relevant question is whether the pupil response at retrieval should be treated as a true memory signal, or whether it simply reflects arousal due to successful recognition, recollection or recall (in the case of free-recall or cued-recall tasks). If the latter is the case, then the pupil response would only reflect subjective memory decisions and not successful memory retrieval. Initial evidence is derived from the recognition memory paradigm, wherein both correct (i.e., hits/correct rejections) and incorrect (i.e., misses/false alarms) responses to old/new stimuli are distinguishable. For instance, it has been observed that pupil dilation is more pronounced for false alarms (new stimuli erroneously identified as old) compared to correct rejections (new stimuli correctly identified as new) [59], suggesting that pupil dilation is sensitive to the subjective recognition of stimuli. Conversely, studies involving amnesic patients [60] have demonstrated a pupil old/new effect despite significantly compromised memory performance. Furthermore, in the study discussed previously [27] the pupil timeseries (change in pupil size over time) differentiated between accurate and inaccurate old/new decisions. Temporally early pupil effects were indicative of the objective status of stimuli, whereas subsequent changes in pupil amplitude reflected the subjectively reported recognition decision. Taken together, pupil response may indicate implicit recognition and may also serve as a signal to distinguish true from false memories.

A more recent study [57], directly explored the pupil response patterns and their potential in distinguishing true memories from false ones, using a false memory paradigm. Specifically, the Deese–Roediger–McDermott (DRM) paradigm was used in two experiments involving either visual or auditorily delivered words. In both experiments, lists of words semantically related to a non-presented critical lure word were used to generate false memories. Pupillometric data were collected at retrieval during which participants encountered studied, non-studied, but related, critical lures and unrelated new words. Three theoretical hypotheses can be proposed regarding the pupillary response’s capacity to distinguish between true and false memories. The first hypothesis posits that pupil dilation merely indicates subjective memory judgments, implying that the old/new effect would manifest in correct recognition as well as in misidentified stimuli, without differentiating between them. In this scenario, the perception of stimuli as old (regardless of their actual familiarity) would uniformly evoke an increase in pupil dilation. This is illustrated in Figure 2b. A second hypothesis suggests that pupillary responses are directly tied to the actual ‘old’ status of stimuli, independent of the subjective memory perception. Here, the pupil dilation is primarily influenced by true rather than false memories. This is illustrated in Figure 2c. A third hypothesis considers that pupillary responses reflect both the objective and the subjective components of memory, indicating that pupil dilation during memory retrieval is influenced by both true and false memories. Nonetheless, the specific patterns which this final hypothesis entails remained undefined. Nevertheless, changes in pupil amplitude across time (e.g., during the recognition period) may be able to capture information processing variations during recognition memory decision.

Indeed, the study by Kafkas et al. [57] showed that pupil responses effectively differentiated true from false memories, with variations in pupil amplitude at different temporal components depending on the memory type that enabled recognition (Figure 2d,e). Specifically, familiarity-based false memories were marked by heightened pupil responses relative to familiarity-based true memories at an earlier stage in the recognition process (Figure 2d). Conversely, recollection-based false memories showed increased pupil dilation compared to recollection-based true memories at a later stage, nearing the end of the recognition period (Figure 2e). Therefore, these findings support the hypothesis that the pupil response reflects retrieval success and not simply arousal due to subjective recognition, and therefore can be used as a true retrieval signal.

### Implications for Theories of False Memory

The pupillometric evidence from this study further suggests that the processes involved in familiarity-based and recollection-based false memories are dissociable. Specifically, the temporal dynamics of pupil dilation can provide insights into the processes that give rise to false memories. The increased pupil dilation for familiarity-based false memories at earlier stages of processing suggests that these types of false memories may be influenced by processes related to perceptual fluency or the ease with which a stimulus is processed [44,61,62,63]. This fluency is enhanced due to the activation of related concepts or words during the encoding phase, which makes the non-presented, but semantically related, critical lures feel more familiar. This aligns with the activation-monitoring framework [64] and the fuzzy-trace theory [65], which despite their theoretical differences, both explain memory illusions as arising from the activation of semantically related or associated items. The increased fluency, resulting from repeated activation of the target concept and the enhancement of the gist, may cause greater pupil dilation compared to stimuli that are truly familiar. Indeed, this heightened fluency is often attributed to familiarity within a recognition memory task [66]. Additionally, the timing of the effect agrees with the widely recognized rapid and automatic characteristics of familiarity-based recognition [67].

The later increase in pupil dilation for recollection-based false memories suggests a more complex and effortful process, possibly involving the construction of a detailed, but incorrect, memory narrative to support a recollection attribution. This could indicate the involvement of monitoring processes [68,69] in attempting to reconcile and make sense of partially retrieved information, leading to the creation of details that were not part of the original event. This process resonates with aspects of the fuzzy-trace theory, which distinguishes between the gist of an event (the overall sense or meaning) and the specific details (verbatim). According to this account, false memories arise from confusion or conflation between the gist and the verbatim elements [65]. Further evidence for this comes from a recent study in which larger pupil dilation was observed during tip-of-the-tongue states, when participants are on the verge of recall compared to when they were not in a tip-of-the-tongue state. Tip of the tongue may indicate enhanced retrieval search and monitoring which may drive the increase in pupil dilation [70]. A similar effect may be expected in freely recalled false memories, where enhanced pupil dilation, particularly during later stages of processing, would suggest more intensive, but erroneous, retrieval search and monitoring processes compared to true recall.

In conclusion, there is compelling evidence for the ability of pupil response to discriminate true from false memories. The results also provide valuable insights into the nature of false memories and their generation across various types of memory. Especially, the temporal dynamics of pupil response during recognition of true and false memories offer a novel understanding of the cognitive processes involved in memory distortions. Beyond the theoretical implications regarding the use of pupillometry as a true measure of successful memory retrieval, the findings reviewed here have significant implications for applied settings. In legal contexts, for example, understanding false memory formation can inform the reliability of eyewitness testimonies. Similarly, in clinical practice, insights into memory distortions can guide rehabilitation approaches for individuals dealing with memory-related disorders. These proposals require further, targeted, investigation.

## 6. Methodological Considerations

Over the past 15 years, pupillometry has made significant contributions to memory research, particularly as more sophisticated experimental designs have been employed. These designs aim not merely to establish descriptive correlations with memory, but critically to test and formulate theories of memory. However, it is important to acknowledge the non-specificity of pupillary response to memory and the various physiological, psychological, and physical factors that may influence these responses. Given these complexities, the following methodological considerations are crucial when designing experiments and interpreting memory-related pupillary effects.

1. Control of cognitive load and extraneous factors: It is essential to meticulously control for cognitive load and the nature of the task during encoding and retrieval phases. Pupillary responses can be influenced by various cognitive processes that are not directly related to memory, including decision-making, arousal, motivation, and task complexity. Therefore, researchers must strive to isolate memory-specific signals from those related to cognitive effort or emotional responses. This distinction is crucial for ensuring the validity of findings concerning memory processes.
2. Hierarchical and systematic manipulation of experimental factors: Expanding on the previous point, it is ideal to systematically manipulate various factors—including experimental variables such as reward, valence, and anticipation—in separate experiments. This approach allows for meticulous examination of their contributions to memory-related pupil responses.
3. Establishing standardized protocols for measuring and interpreting pupil size changes across various research settings is crucial. This involves standardizing the timing of measurements, environmental conditions, and data analysis techniques (see e.g., [71]). Special emphasis should be placed on selecting appropriate baseline conditions to enable the calculation of trial-specific pupillary responses. Baseline conditions should be implemented on a trial-by-trial basis and must effectively control or eliminate luminance effects. Additionally, the sensitivity of pupil responses to psychological factors such as anticipation and surprise should be considered during baseline selection. For instance, using a mask composed of scrambled pixels from a target image could inadvertently increase pupil dilation due to the unexpected nature of the arrangement (unpublished observation shown in [72]). This underscores that not all stimulus types are suitable for pupillometry. Baseline selection should be tailored to the specific stimuli used in an experiment.
4. External and internal context: It is imperative that both external and individual-specific internal conditions be controlled with greater rigor than in other experimental methods. The external context encompasses factors such as lighting conditions, stimulus characteristics, and timing. Internal states, including fatigue, the influence of chemical substances (such as caffeine and alcohol), and medication, must also be carefully considered. For additional methodological considerations see [73].
5. Integration with other neurophysiological measures: Combining pupillometry with other neurophysiological measures such as EEG or fMRI could provide a more comprehensive understanding of the neural networks underlying memory processes. Furthermore, many models proposed to explain the pupillary response to various cognitive states and memory, as discussed in this review, suggest neurocognitive explanations that necessitate pharmacological, neurophysiological and neuroimaging investigations. Therefore, this represents a critical direction for future research as pupillometry by itself only provides an indirect measure of brain activity and cognitive processing. Such a multimodal approach can help validate findings and elucidate the interplay between autonomic responses and brain activity in the service of memory processes.

## 7. Conclusions and Future Directions

Pupillometry has experienced a resurgence over the last 15 years as a significant measure of cognition in experimental psychology and neuroscience. This review underscored its substantial contributions to the study of episodic memory, expanding beyond its traditional role as a measure of effort and cognitive load. The evidence reviewed indicates that with careful experimental considerations, pupil response can significantly enhance our understanding of the processes underlying memory encoding and retrieval. Meticulous experimental design, along with the application of pupillometry across various groups and its integration with other methodologies, has the potential to profoundly inform and test theories of memory formation and retrieval.

Considering emerging trends, it can be speculated that pupillometry-based memory research will benefit from cross-sectional or even longitudinal studies that trace the development of memory systems from infancy through late adulthood, covering both normal and abnormal aging. These studies have the potential to influence educational practices and the early diagnosis of memory impairments. Additionally, utilizing pupillometry in clinical settings to assess memory dysfunction in conditions such as Alzheimer’s disease and other dementias could serve as a non-invasive biomarker for early diagnosis and ongoing monitoring (see also [74]). Finally, applying machine learning and advanced computational techniques to pupillometry data may enable more accurate real-time assessments of cognitive/memory states in clinical and legal contexts. However, this approach requires further systematic investigation to validate its effectiveness.

## Data Availability

No new data were created or analyzed in this study. Data sharing is not applicable to this article.
